# Supplementary material for: Asymmetric adhesion of rod-shaped bacteria controls microcolony morphogenesis
Source: Nat Commun. 2018 Mar 16;9:1120. doi: 10.1038/s41467-018-03446-y (PMC5856753; doi:10.1038/s41467-018-03446-y)
Supplement: Supplementary file 1 — Supplementary Information(PDF 9346 kb) [file 41467_2018_3446_MOESM1_ESM.pdf]

Asymmetric adhesion of rod-shaped bacteria controls microcolony morphogenesis

Marie-Cécilia Duvernoy[1,2], Thierry Mora[1], Maxime Ardré[1], Vincent Croquette[1], David Bensimon[1,3], Catherine Quilliet[2], Jean-Marc Ghigo[4], Martial Balland[2], Christophe Beloin[4], Sigolène Lecuyer[2], Nicolas Desprat[1,5,*]

1 Laboratoire de Physique Statistique, École Normale Supérieure, PSL Research University; Université Paris Diderot Sorbonne Paris-Cité; Sorbonne Universités UPMC Univeristé Paris 06; CNRS; 24 rue Lhomond, 75005 Paris, France.
2 Université Grenoble Alpes - CNRS, LIPHY, F-38000 Grenoble, France.
3 Deptartement of Chemistry and Biochemistry, UCLA, Los-Angeles, CA, USA.
4 Genetics of Biofilms, Institut Pasteur, rue du Dr. Roux. 75015 Paris, France.
5 Paris Diderot University, 10 rue Alice Domon et Leonie Duquet. 75013 Paris, France.

***Corresponding author (desprat@ens.fr)**

Supplementary Figures

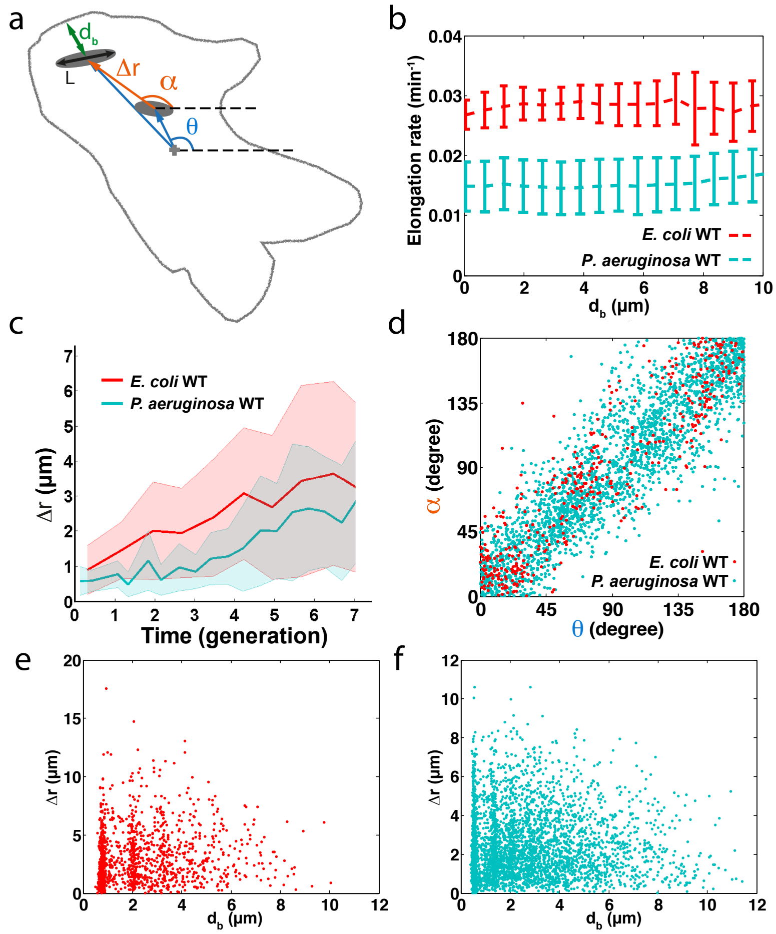

Supplementary Figure 1. Bacterial elongation drives microcolony expansion. (**a**) Parameters used to quantify cell movements occurring during each cell cycle: cell length (L), average distance to the edge of the colony d_b, angular position averaged over the cell cycle in the microcolony (θ), angular direction (α) and magnitude (Δr) of cell movement from the beginning to the end of the cell cycle. (**b**) The elongation rate $\frac{1}{L}\frac{dL}{dt}$ is uniform inside the microcolony. (**c**) Average cell displacement Δr as a function of time from the beginning of microcolony growth for WT *E. coli* and *P. aeruginosa* PAO1. Δr of a given cell is computed as the distance travelled between birth and cell division. (**d**) Correlation between orientation of individual cell movement α and the angular position θ of bacteria.(**e,f**) Displacement Δr of bacteria between their birth and division as a function of their distance d_b to the colony edge. Each point represents a cell, and data from different microcolonies are superimposed. In (**e**), WT *E.coli* (N=7 microcolonies, red). In (**f**), WT *P. aeruginosa* (N=10 microcolonies, cyan). In all panels, data are plotted in red for WT *E. coli* (N=7 microcolonies) and in cyan for WT *P. aeruginosa* PAO1 (N=10 microcolonies). Error bars are defined as s.d.. Microcolonies were monitored up to the second layer formation.

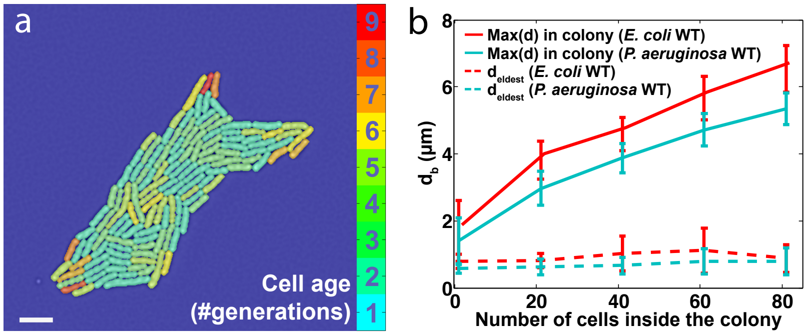

Supplementary Figure 2. The oldest bacteria are located at the periphery. (**a**) Distribution of cell age within a WT *E. coli* microcolony. Colors code for the age of bacteria given by the history of their oldest pole (blue is young; red is old). Scale bar represents $5\mu m$. (**b**) Distance of the oldest cell from the edge of the microcolony (dashed line) as a function of the number of cells in the microcolony. Solid line displays the characteristic size of the microcolony, computed as the maximal distance to the edge that can be measured for each cell inside the colony. Error bars are defined as s.d..

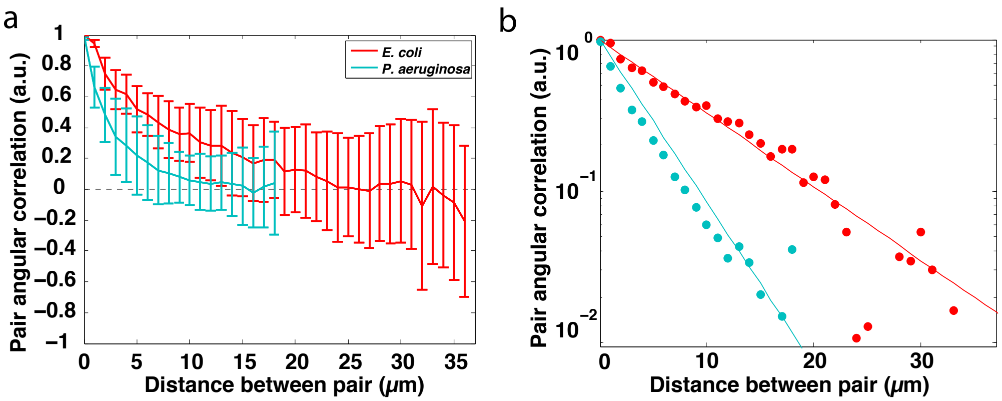

Supplementary Figure 3. Bacterial orientation inside the microcolony. (**a**) The nematic order parameter in 2D $\langle 2cos^2(\theta_{i,j}) - 1 \rangle_{i,j}$ as a function of the distance between two bacteria for microcolonies counting around 100 cells ($\theta_{i,j}$ is the angle between bacteria i and j). The same graph is plotted in log-lin scale in (**b**) WT *E.coli* (N=7 microcolonies, red); right, WT *P. aeruginosa* (N=10 microcolonies, cyan). Error bars are defined as s.d..

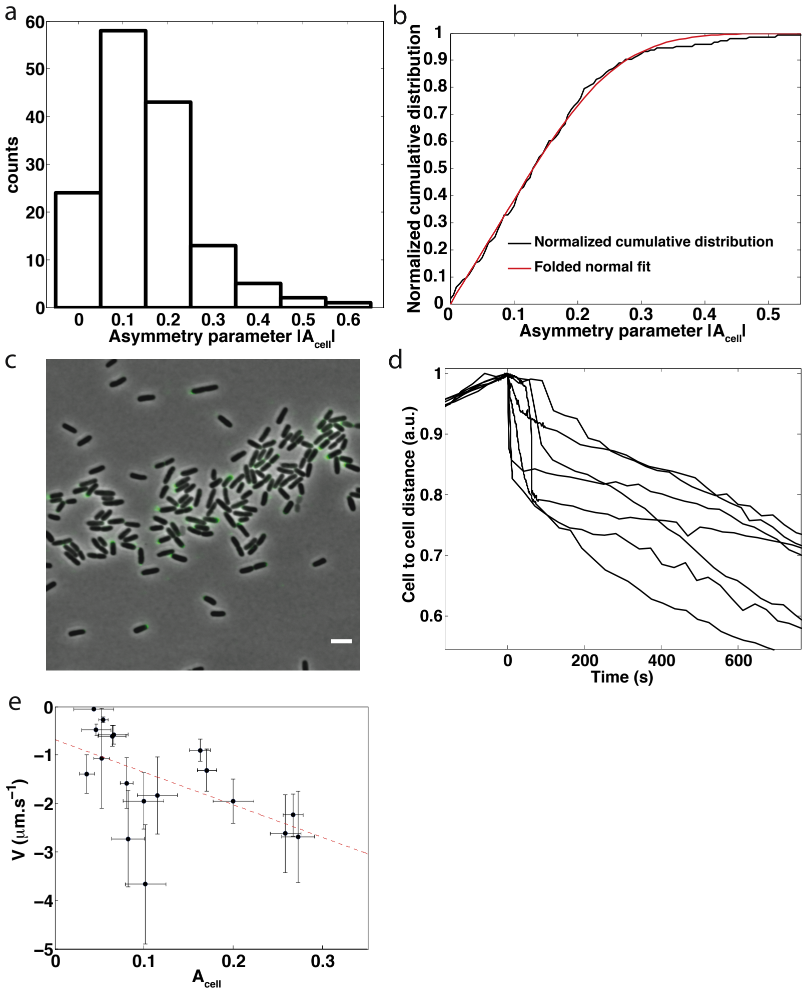

Supplementary Figure 4. Asymmetric adhesion induces reorganization of the two daughter cells after division. (**a**) Histogram of | A_{cell} | for a wild type population of *E. coli* cells. (**b**) Normalized cumulative distribution (black line) of the histogram shown in A is fitted with normalized cumulative distribution of a folded normal law (black line). (**c**) Immunodetection of Ag43 in a WT *E. coli* culture started from a colony expressing Ag43, using anti-Ag43 polyclonal rabbit antiserum raised against the α-domain of Ag43. Because expression of ag43 is bistable [1], not all bacteria express Ag43. Yet, for the fraction of cells that express it, Ag43 is localized only at one pole. Scale bar represents $5\mu m$. (**d**) Distance between the CM of the two daughter cells normalized by the size at division. Before division, cell-to-cell distance is computed as half of total cell length. Traces show individual reorganizations. Time is set to 0 when the cell to cell distance is maximal. (**e**) Magnitude of the reorganization of the two daughter cells as a function of the mother cell asymmetry A_{cell}. Magnitude of the reorganization is computed as the maximal derivative of cell-cell distance measured for 100 s after this distance has reached its maximum. The sign of reorganization is negative, since the two daughter cells are moving closer. For comparison, the sliding speed due to cell elongation is on the order of $5nm.s^{-1}$.

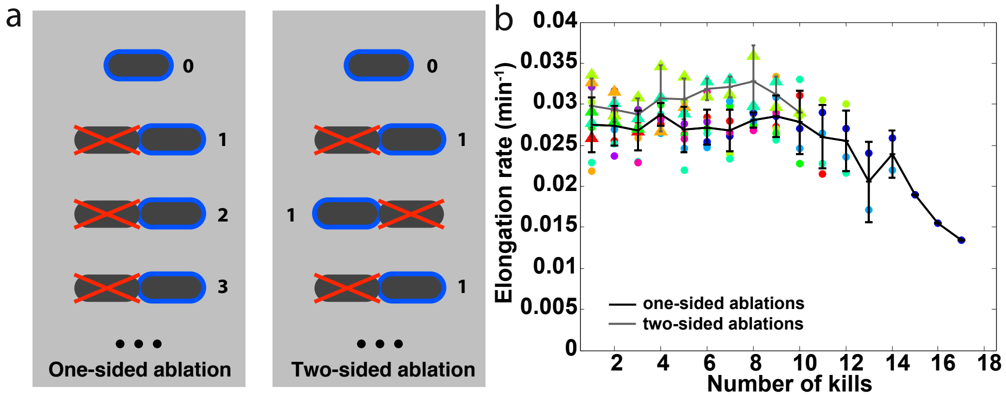

Supplementary Figure 5. One-sided and two-sided ablations. (**a**) For one-sided ablations, we systematically ablated the left daughter cell after division. Therefore, the oldest pole of the remaining cell ages throughout successive rounds of ablation. For two-sided ablations, ablations were alternated between the left and right daughter cell at each generation. Therefore, the oldest pole of the remaining cell was never older than one generation throughout successive rounds of ablations (except for the first two rounds). Numbers illustrate age of the oldest pole, which starts arbitrarily at 0. Red cross indicates the ablated cell. Time in generations (i.e. one cell cycle) goes from top to bottom. (**b**) The elongation rate of the remaining cell as a function of the number of cell cycles during the course of one-sided ablation (black line) and two-sided (gray line) experiments. Error bars are defined as s.d.. The different points represent individual experiments corresponding to one-sided (dots) and two-sided ablations (triangles).

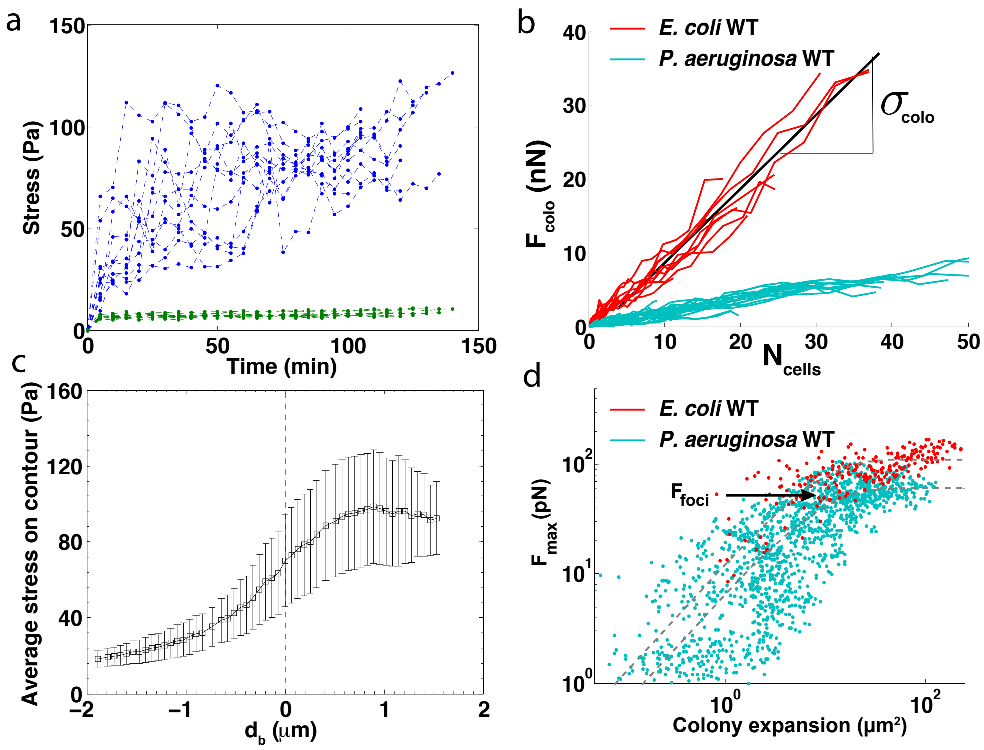

Supplementary Figure 6. Stress beneath and outside of the microcolony.. (**a**) Time evolution of stress beneath (blue) and outside (green) microcolonies of WT *E. coli*. (**b**) Total sum of force magnitudes, F_{colo}, developed by independent microcolonies on the substrate increases linearly with the area of the microcolony for both WT *E. coli* (red) and WT *P. aeruginosa* (cyan). We compute stress σ_{colo} beneath the microcolony as the average slope of these curves. (**c**) Average adhesive stress as a function of distance d_b to the edge of WT *E. coli* microcolonies. Microcolonies are sliced in concentric contours going from the edge to the center. Based on stress maps shown in Fig. 3, we measured mean stress inside the contour computed as the integral of the stress along the contour divided by the length of the contour. The edge of the colony is set at $d_b = 0$. The inside of the microcolony corresponds to $d_b > 0$. Error bars are defined as s.d. of stress distribution in concentric contours. (**d**) Maximal local force measured at adhesion foci in the colony saturates early during growth for WT *E. coli* (red) and WT *P. aeruginosa* (cyan). The force at adhesive foci, F_{foci}, corresponds to the value of the plateau.

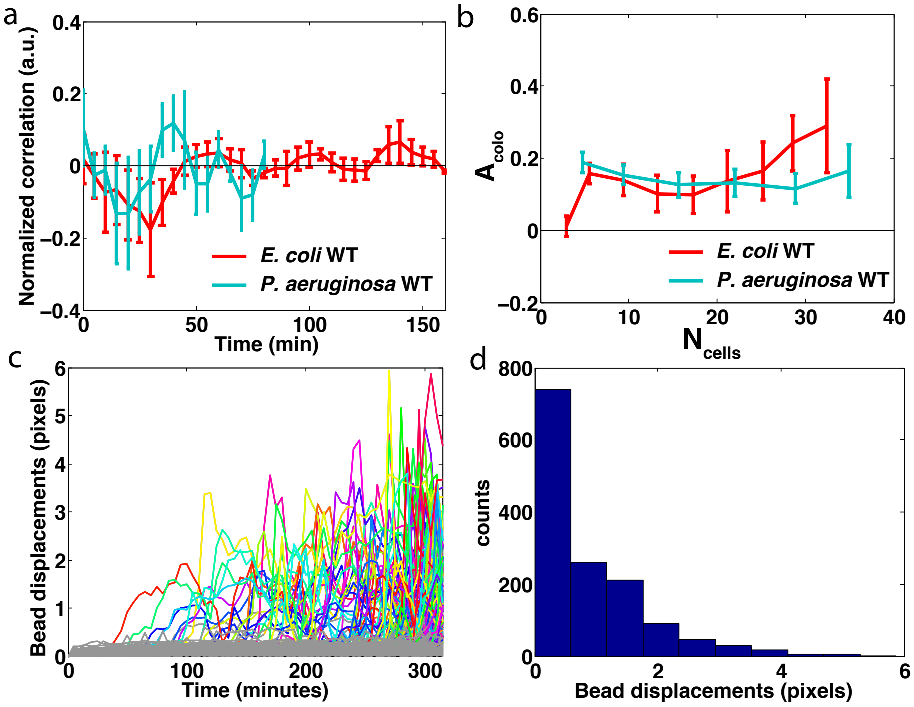

Supplementary Figure 7. Rupture of adhesive bonds. (**a**) Average correlation between pole displacement and force variation in wild type microcolonies of *E. coli* (red, N=12) and *P. aeruginosa* (cyan, N=16). Poles are tracked from birth and span several generations (example of an individual trace is shown in Fig. 3e).(**b**) Force asymmetry of microcolony A_{colo} for WT *E. coli* (red, N=12) and WT *P. aeruginosa* (cyan, N=16) measured as $A_{colo} = [max(F_{old}) - max(F_{new})]/F_{foci}$. (**c**) Displacement of individual beads located in the PAA gel under the colony (colors) and outside the colony (gray). Time 0 refers to the start of the experiment. All individual trajectories from individual microcolony growth are displayed. Displacements are computed as the distance between the position of the bead at a given time relative to its position on the first frame. (**d**) Histogram of bead displacements for beads located inside the PAA gel under the microcolony. Data from all time points shown in (c) are pooled in the distribution.

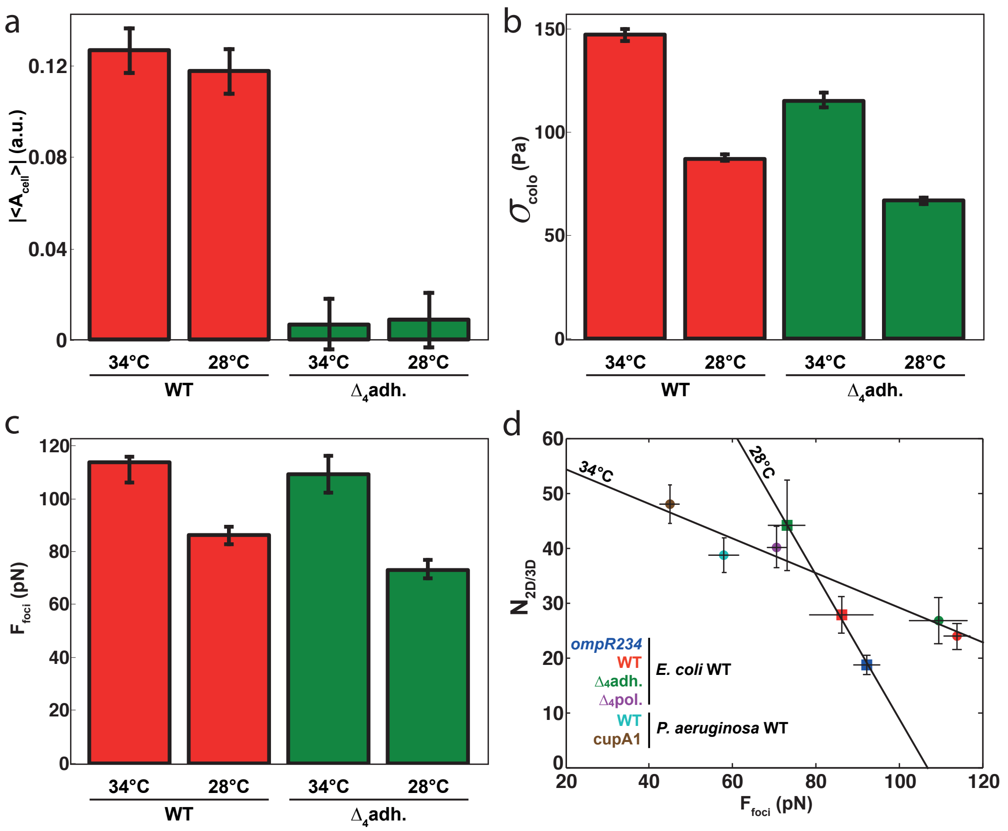

Supplementary Figure 8. Direct comparison of adhesive properties at 28°C and 34°C. (**a**) Average asymmetry $| \langle A_{cell} \rangle |$ of populations of isolated bacteria for WT *E. coli* (red, N=142 for 28°C, N=146 for 34°C), of a mutant deleted for four adhesins $\Delta_4$adh, Δ*fliER_agn43_fimAH_csgA* (green, N=115 for 28°C, N=95 for 34°C). σ_{colo} (**b**) and F_{foci} (**c**) measured by force microscopy experiments for WT *E. coli* (red, N=13 for 28°C, N=12 for 34°C) and a mutant deleted for four adhesins $\Delta_4$adh, Δ*fliER_agn43_fimAH_csgA* (green, N=7 for 28°C, N=19 for 34°C). (**d**) Number of bacteria at the onset of second layer formation as a function of the force at adhesion foci for all strains considered in this study at 28°C and 34°C.

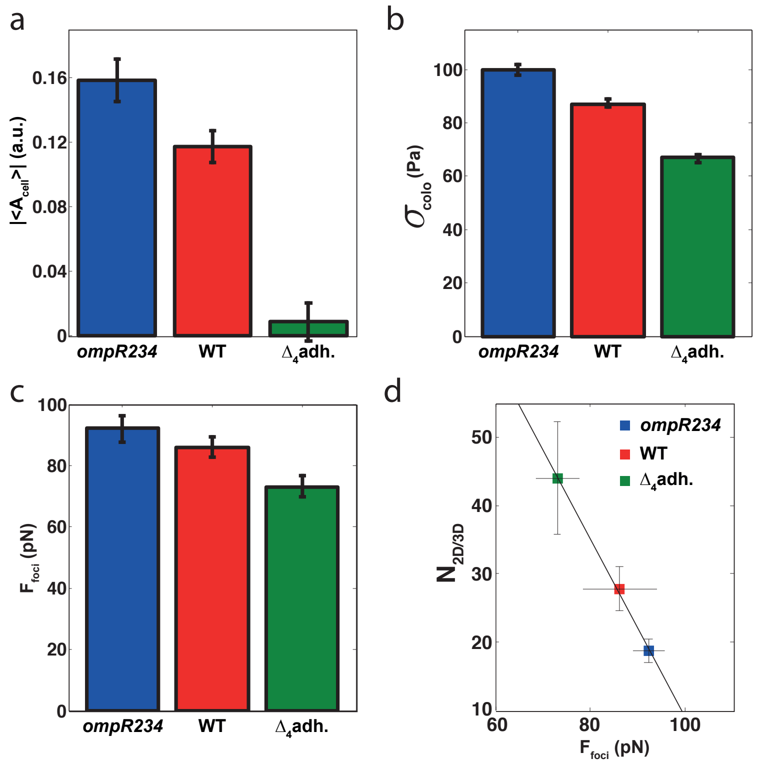

Supplementary Figure 9. Cell-substrate adhesion at 28°C. (**a**) Average asymmetry $\mid \langle A_{cell} \rangle \mid$ of populations of isolated bacteria for WT *E. coli* (red, N=142), of a mutant deleted for four adhesins $\Delta_4$adh, Δ*fliER_agn43_fimAH_csgA* (green, N=115) and of *ompR234* mutant (blue, N=82) which over-expresses curli fibers. σ_{colo} (**b**), F_{foci} (**c**) and number of bacteria at the onset of second layer formation as a function of force at adhesion foci (**d**) measured by force microscopy for WT *E. coli* (red, N=13), a mutant deleted for four adhesins $\Delta_4$adh, Δ*fliER_agn43_fimAH_csgA* (green, N=7) and *ompR234* mutant (blue, N=10) that over-expresses curli fibers.

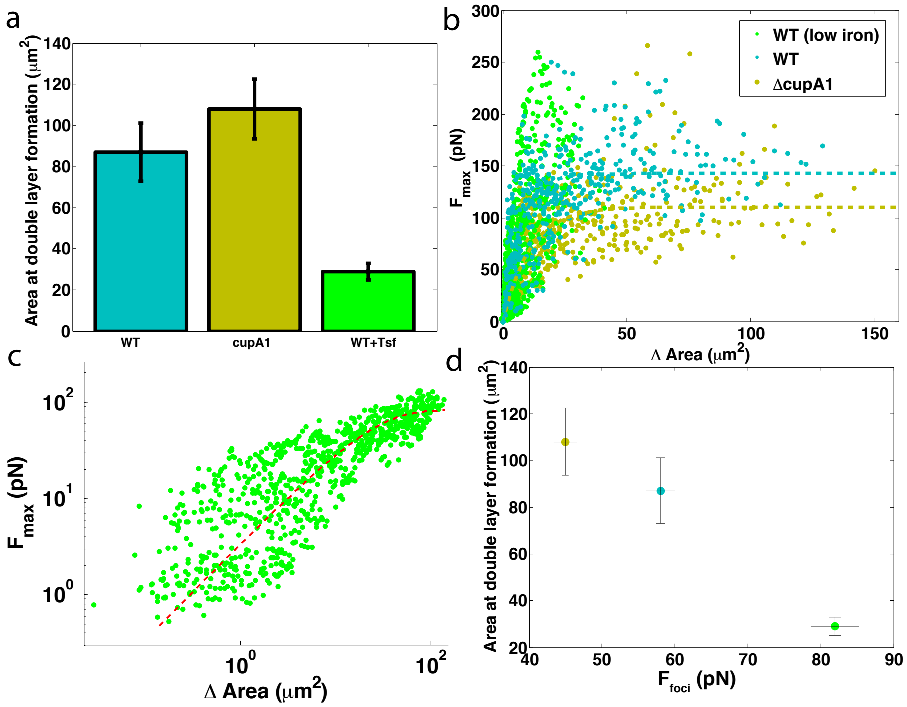

Supplementary Figure 10. Force microscopy under low iron conditions. To reduce the availability of iron in the environment, we added Transferrin (Tsf) to succinate minimal medium (SMM). (**a**) Microcolony size at the onset of second layer formation for WT *P. aeruginosa* (cyan, N=10) and cupA1-*P. aeruginosa* (brown, N=15) in normal conditions (LB); WT *P. aeruginosa* in low iron conditions (SMM with Tsf) (green, N=12). (**b**) Force measured at adhesion foci for the same set of strains and conditions. The microcolonies are tracked up to formation of second layer. The force at adhesion foci for WT *P. aeruginosa* under low iron conditions displays a rapid increase from the beginning of growth. (**c**) To quantify the value of the plateau under low iron conditions, we analyzed the force at adhesion foci after formation of second layer. We found that the value of the plateau under low iron conditions is significantly higher than in normal conditions. (**d**) Relation between the force in adhesion foci and size of the microcolony at the onset of the second layer.

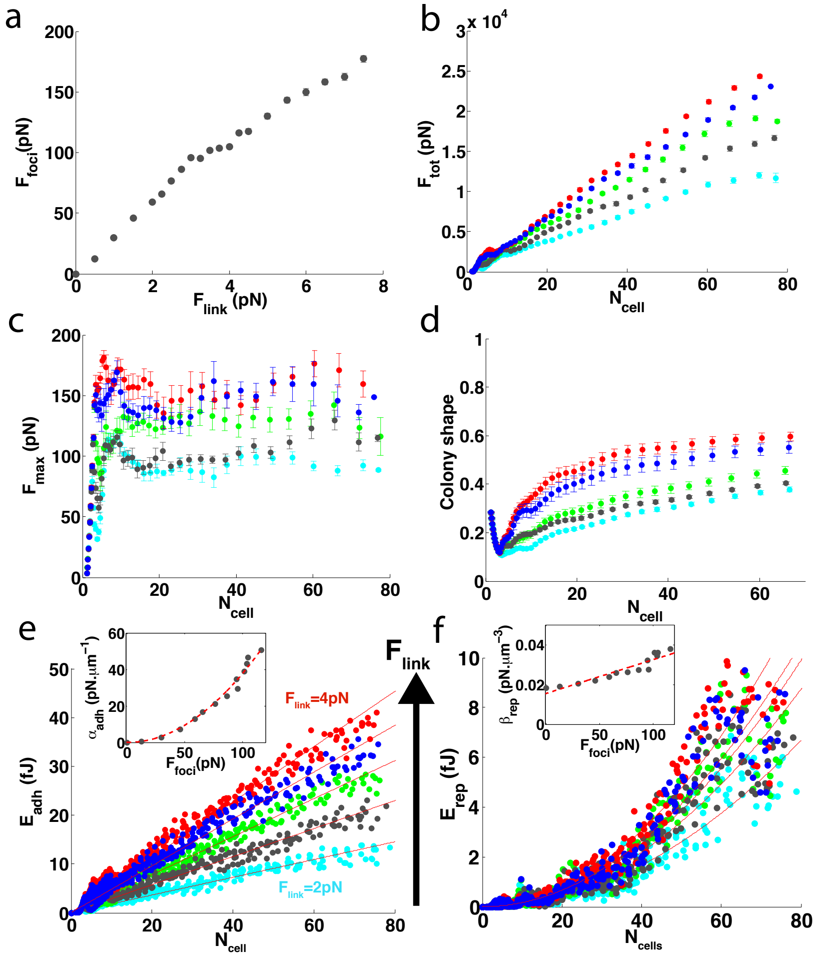

Supplementary Figure 11. Simulation of microcolony morphogenesis. (**a**) Relationship between force at adhesion foci F_{foci} and rupture force F_{link} of a single adhesive link. (**b**) F_{tot} as a function of colony size. (**c**) Maximal force F_{max} as a function of colony size. We used these curves to fit the value of plateau F_{foci} for simulations. (**d**) Aspect ratio for different microcolonies. (**e**) Numerical simulations show that the adhesive energy stored in the elastic links linearly increases with the number of bacteria in the microcolony $E_{adh} = \alpha_{adh} N_{cells}$; the prefactor α_{adh} quadratically scales with the saturation force (inset). (**f**) The repulsive energy between cells quadratically increases with the number of bacteria in the microcolony $E_{rep} = \beta_{rep} N_{cells}^2$; pre-factor β_{rep} linearly increases with the saturation force (inset). In all panels, plots represent the average of 10 simulations. Colors correspond to different values of F_{link} at which adhesive links rupture (red, 4pN; blue, 3.5pN; green, 3pN; gray, 2.5pN; cyan, 2pN).

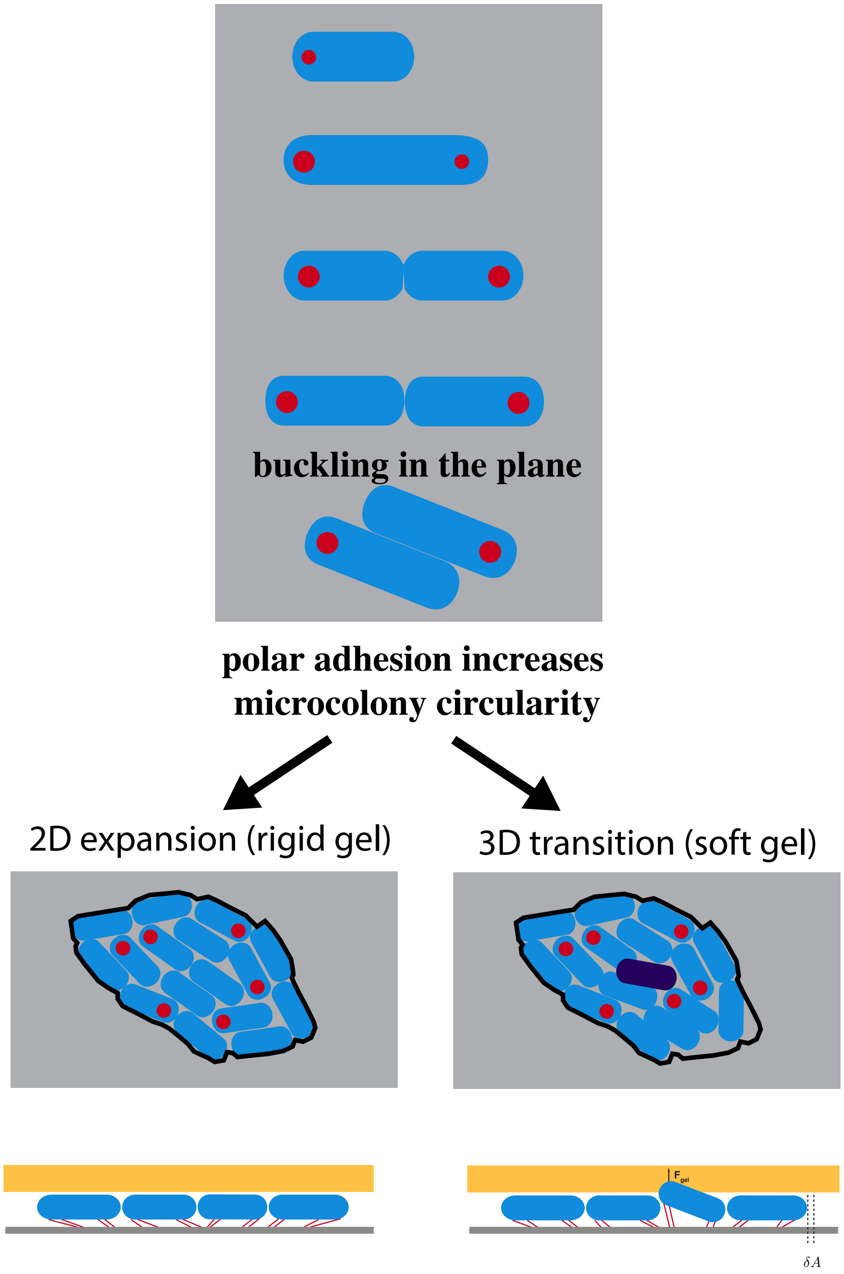

Supplementary Figure 12. Schematic of microcolony morphogenesis. Polar adhesion forces daughter cells to slide alongside each other, and subsequently increases circularity of the microcolony. On soft gel, bacteria form second layers at smaller microcolony size than on rigid gel. The black boundary depicted in the two cases is the same. Red points are used to schematize polar adhesion. The cell highlighted in purple is the cell that starts to grow on top of the others.

Supplementary Tables

Supplementary Table 1. Strain description

Strains	Relevant genotypic and phenotypic characteristics	Source or reference
E. coli		
WT	MG1655 F-, λ-, rph-1	*E. coli* genetic stock center CGSC#6300
$\Delta_4$adh	MG1655_*gfp*_Δ*fliER*::*cm*_Δ*fimAH*::*zeo*_Δflu::FRT_Δ*csgA*::*spec*, deletion mutant of flagella, type 1 fimbriae, Ag43 and curli	[2]
$\Delta_4$pol	MG1655_Δ*yjbEH*::*cm*_Δ*bcsA*::*Km*FRT_Δ*pgaA*::*uidA*-*zeo*_*cps5*::Tn10, deletion mutant of polysaccharides Yjb, cellulose, PGA and colanic acid. Δ*bcsA*::*Km*FRT comes from Keio collection strain JW5665 [3]. *cps5*::Tn10 comes from [4]	Gift from Bianca Audrain
ompR234	MG1655_*gfp*_*ompR234*_*malA*::*Km*, constitutive expression of curli genes	[5]
P. aeruginosa		
WT	PAO1 [6]	Gift from Isabelle Schalk
WT	PA14	Gift from Kolter lab
cupA1	*cupA1*::MrT7, deletion mutant of fimbriae. MrT7 comes from transposon mutant library [7]	Gift from Frederick Ausubel

Supplementary Table 2. Primers used in this study to construct deletion mutants

Mutation constructed		
Δ*yjbEH*::*cm*	tttactcagggcgtgatcctgaac	yjbE.ext-5
	acctccttcacagtggcgagcttc	yjbH.ext-3
	gaaatcagcagccacgatag	yjbE.500-5
	tggatttggagcgcgcctccac	yjbH.500-3
	gaagtgatcttccgtcacagcataacaatttcccttcattgaatg	yjbE.catL-3
	gagtggcagggcggggcgtaagtcatgggaaaggtgccagttttc	yjbH.CatL-5
Δ*pgaA*::*uidA*-*zeo*	caaagcaagcccaaacaataag	*pgaA*.ext-5
	cgtagtgagaattccatgtatg	*pgaA*.ext-3
	cagtactactaattctcaaa	*pgaA*.500-5
	gagctcaacgagccgggaac	*pgaA*.500-3
	ttgccaaaatctctctctgtatctaattacaggtaactgaaaagaaag	*pgaA*.uidA.L-3
	ttcgtggccgaggagcaggactgaggataaatatgttacgtaatggaa	*pgaA*.uidA.L-5

Supplementary Table 3. Description of the different experimental parameters

Name	Description
N_{cells}	Number of bacteria in the microcolony
$N_{2D/3D}$	Number of bacteria in the microcolony at second layer formation
$a(m)$	Length of the major axis of the ellipse that fits the mask of the microcolony
$b(m)$	Length of the minor axis of the ellipse that fits the mask of the microcolony
$\Delta X(m)$	Displacement of the center of mass of a single cell
$\Delta L(m)$	Cell elongation
A_{cell}	Level of asymmetry in single bacteria adhesion
A_{colo}	Global level of asymmetry in microcolonies
$\sigma_{colo}(Pa)$	Stress generated by the microcolony
$F_{foci}(N)$	Force at adhesion foci in the microcolony
$F_{old}(N)$	Adhesion force at an individual old pole
$F_{young}(N)$	Adhesion force at an individual young pole
$d(m)$	Distance traveled by a pole since its apparition
$d_b(m)$	Distance between bacteria and the border of the microcolony
$\Delta r(m)$	Distance traveled by a bacteria over one cell cycle
$\Delta\alpha(rad)$	Angle between the horizontal and the direction of cell displacement
$\Delta\theta(rad)$	Angular position of bacteria within the microcolony with respects to the horizontal

Supplementary Table 4. Description of the different parameters used in the model

Name	Value	Description	Reference
dt	2s	Time increment	
g	$1.6h^{-1}$	Growth rate	*This study*
d_c		Cell length	
r_0	$1.4\mu m$ (*coli*); $0.9\mu m$ (*aeruginosa*)	Cell width	*This study*
d_L	$8.8\mu m$ (*coli*); $4.6\mu m$ (*aeruginosa*)	Cell length at which cell may divide	*This study*
k_{rep}	$10^4 pN.\mu m^{-1}$	Elastic constant for cell repulsion	[2]
V_0	$2.5pN.\mu m^{-1}$	Depth of the attractive potential	
r_1	$0.1\mu m$	Attraction length	
k_{on}	$22.5min^{-1}$	Rate of adhesive link formation per cell	
k_{off}	$10^{-2}min^{-1}$	Rate of adhesive link loss	
n_l	250	Maximum links per cell	
L_p	0.828nm	Persistence length	[8]
L_0	$10\mu m$	Length of a fully extended link	[9]
F_{link}	few pN	Rupture force of a single adhesive link	[10]
η	$2pN.s.\mu m^{-2}$	Coefficient of the viscous friction with the walls	
E_0	4kPa	Young modulus of the above gel	*This study*
ν_0	0.5	Poisson ratio of the above gel	
z	$0.2\mu m$	Vertical indentation of the gel at second layer formation	
γ	0.1	Coefficient used to compute the vertical work of adhesive forces	

Supplementary References

[1] Chauhan, A., Sakamoto, C., Ghigo, J.-M. & Beloin, C. Did I pick the right colony? Pitfalls in the study of regulation of the phase variable antigen 43 adhesin. *PloS One* **8**, e73568 (2013).

[2] Francius, G. *et al.* Bacterial surface appendages strongly impact nanomechanical and electrokinetic properties of Escherichia coli cells subjected to osmotic stress. *PloS One* **6**, e20066 (2011).

[3] Baba, T. *et al.* Construction of Escherichia coli K-12 in-frame, single-gene knockout mutants: the Keio collection. *Molecular Systems Biology* **2**, 2006.0008 (2006).

[4] Parker, C. T. *et al.* Role of the rfaG and rfaP genes in determining the lipopolysaccharide core structure and cell surface properties of Escherichia coli K-12. *Journal of Bacteriology* **174**, 2525–2538 (1992).

[5] Beloin, C., Houry, A., Froment, M., Ghigo, J.-M. & Henry, N. A short-time scale colloidal system reveals early bacterial adhesion dynamics. *PLoS biology* **6**, e167 (2008).

[6] Stover, C. K. *et al.* Complete genome sequence of Pseudomonas aeruginosa PAO1, an opportunistic pathogen. *Nature* **406**, 959–964 (2000).

[7] Liberati, N. T. *et al.* An ordered, nonredundant library of Pseudomonas aeruginosa strain PA14 transposon insertion mutants. *Proceedings of the National Academy of Sciences of the United States of America* **103**, 2833–2838 (2006).

[8] Rief, M., Oesterhelt, F., Heymann, B. & Gaub, H. E. Single Molecule Force Spectroscopy on Polysaccharides by Atomic Force Microscopy. *Science (New York, N.Y.)* **275**, 1295–1297 (1997).

[9] Forero, M., Yakovenko, O., Sokurenko, E. V., Thomas, W. E. & Vogel, V. Uncoiling mechanics of Escherichia coli type I fimbriae are optimized for catch bonds. *PLoS biology* **4**, e298 (2006).

[10] Liang, M. N. *et al.* Measuring the forces involved in polyvalent adhesion of uropathogenic Escherichia coli to mannose-presenting surfaces. *Proceedings of the National Academy of Sciences of the United States of America* **97**, 13092–13096 (2000).
