# Supplementary material for: Asymmetric adhesion of rod-shaped bacteria controls microcolony morphogenesis
Source: Nat Commun. 2018 Mar 16;9:1120. doi: 10.1038/s41467-018-03446-y (PMC5856753; doi:10.1038/s41467-018-03446-y)
Supplement: Supplementary file 3 — Description of Additional Supplementary Information(PDF 91 kb) [file 41467_2018_3446_MOESM3_ESM.pdf]

Description of Additional Supplementary Files

File Name: Supplementary Movie 1
Description: **WT *E. coli* microcolony grown between agarose and glass.** Phase contrast images were taken every 3min. Time is displayed in hours and minutes. Scale bar 10µm.

File Name: Supplementary Movie 2
Description: $\Delta_4$adh *E. coli* **microcolony grown between agarose and glass.** Phase contrast images were taken every 3min. Time is displayed in hours and minutes. Scale bar 10µm.

File Name: Supplementary Movie 3
Description: **Asymmetric adhesion during the first cell cycle in** *E. coli*. Phase contrast images were taken every 3min. Arrows depict initial position of the cell. Time is displayed in minutes. Scale bar 5µm.

File Name: Supplementary Movie 4
Description: **Single cell ablations for dividing** *E. coli*. Correlation images were taken every 3min. After each division, ablation was performed either on the same side as the previous one (one-sided mode, movie on the left) or on the side opposite to the previous one (two-sided mode, movie on the right). In one-sided mode, the pole of the remaining daughter bacterium is aging. In contrast, it is rejuvenating after each division in the two-sided mode. Red and blue dots, respectively, indicate new and old poles. Time is displayed in hours and minutes. Scale bar 3µm.

File Name: Supplementary Movie 5
Description: **Reorganization of the two daughter cells after division in** *E. coli.* Phase contrast images were taken every second after the septum was visible. Time is displayed in minutes and seconds. Scale bar 3µm.

File Name: Supplementary Movie 6
Description: **Force microscopy.** Map of the stress that an *E. coli* microcolony exerts on the polyacrylamide gel. Images were taken every 3min. Blue and red in the color code corresponds, respectively, to 0Pa and 200Pa. Time is displayed in hours and minutes. Scale bar 5µm.

File Name: Supplementary Movie 7
Description: **Map of forces.** The map of forces that bacteria exert on the polyacrylamide gel for the microcolony shown in supplementary movie 6. Force vectors are displayed in red. Images were taken every 3min.

File Name: Supplementary Movie 8
Description: **Simulation of stress transmitted to the substrate during microcolony growth.** Stress exerted on the substrate is colored from blue (0Pa) to red (200Pa). For this particular simulation, F_{link} = 4pN.

File Name: Supplementary Movie 9
Description: **Simulation of microcolony growth.** Movie of bacteria and adhesive links for the simulated microcolony shown in supplementary movie 8. Bacteria are shown in light gray. Adhesive links are drawn in green and stretch, between the pole the bacteria and the substrate, where they attached when they were first exposed on the cell wall.
